# Willingness of Adults in the United States to Receive HIV Testing in Dental Care Settings: Cross-Sectional Web-Based Study

**DOI:** 10.2196/17677

**Published:** 2020-07-21

**Authors:** Matthew T Rosso, Akshay Sharma

**Affiliations:** 1 Center for Sexuality and Health Disparities University of Michigan School of Nursing Ann Arbor, MI United States; 2 Department of Health Behavior and Biological Sciences University of Michigan School of Nursing Ann Arbor, MI United States

**Keywords:** HIV testing, HIV prevention, dental care settings

## Abstract

**Background:**

The Centers for Disease Control and Prevention estimates that 1.1 million people in the United States are living with HIV and 1 in 8 are estimated to be unaware of their serostatus. Little is known about whether individuals would consider being tested for HIV in nontraditional health care settings such as a dentist’s office. Studies in selected US cities have indicated high acceptability of receiving an HIV test among people attending dental clinics. However, we are not aware of studies that have assessed willingness to receive HIV testing in dental care settings at a national level.

**Objective:**

Using a web-based sample of adult residents of the United States, we sought to assess the self-reported willingness to receive any type of HIV testing (ie, oral fluid rapid testing, finger-stick blood rapid testing, or venipuncture blood testing) in a dental care setting and evaluate independent associations of willingness with the extent to which dental care providers were perceived as knowledgeable about HIV and how comfortable participants felt discussing HIV with their dental care providers.

**Methods:**

Participants were recruited using banner advertisements featured on social networking platforms (Facebook and Instagram) from December 2018 to February 2019. Demographic and behavioral data including information on sexual behaviors in the past 6 months, HIV testing history, and dental/health care–seeking history were collected using an anonymous web-based survey. Willingness to receive any type of HIV testing in a dental care setting was assessed on 4-point scale from very willing to very unwilling. Factors independently associated with participants’ willingness were identified using a multivariable logistic regression model.

**Results:**

Of the 421 participants in our study aged 18 to 73 years, 271 (64.4%) reported having oral sex, 197 (46.8%) reported having vaginal sex, and 136 (32.3%) reported having anal sex in the past 6 months. Approximately one-third had never been tested for HIV (137/421, 32.5%), and the same proportion had not been tested in the past year (137/421, 32.5%). Most participants had dental insurance coverage (356/421, 84.6%), and more than three-fourths reported being very or somewhat willing (326/421, 77.4%) to receive any type of HIV testing in a dental care setting. Higher levels of willingness were associated with being 18 to 24 years versus ≥35 years (aOR 3.22, 95% CI 1.48-6.98), 25 to 34 years versus ≥35 years (aOR 5.26, 95% CI 2.52-10.98), believing that one’s dental care provider is knowledgeable about HIV (aOR 2.04, 95% CI 1.06-3.92), and feeling comfortable discussing HIV with one’s dental care provider (aOR 9.84, 95% CI 3.99-24.27).

**Conclusions:**

Our data indicate high acceptability of receiving HIV testing in a dental care setting, especially among those who report having a positive patient-provider relationship. Future research should focus on assessing dental care providers’ attitudes, self-efficacy, and beliefs about whether HIV testing fits into the scope of dentistry.

## Introduction

The US Centers for Disease Control and Prevention (CDC) estimates that 1.1 million people in the United States are living with HIV and that 1 in 8 are unaware of their serostatus [1]. Decreasing the number of HIV-positive persons who are unaware of their infection is critical to advancing HIV prevention efforts [2]. The US Preventive Services Task Force recommends that clinicians screen all adolescents and adults aged 15 to 65 years at least once in their lifetime in order to identify those who are HIV positive and repeatedly screen those who are known to be at risk for HIV infection, those who actively engage in risky behaviors, and those who live in or receive medical care in high-prevalence settings [3]. Although HIV testing levels in the general US population have increased over time (from 38% in 2005 [4] to 46% in 2017 [5]), more than half of all nonelderly Americans report never having been tested for HIV [5].

Rapid HIV testing using an oral fluid specimen (20-minute test) was approved by the US Food and Drug Administration (FDA) for professional use in 1996 [6] and for home use in 2012 [7]. Similarly, rapid HIV testing using a finger-stick blood specimen (1-minute test) was approved for professional use by the FDA in 2015 [8]. Given the ease of oral fluid and finger-stick blood specimen collection and the short wait time for test results, it is worth exploring nontraditional settings in which such tests could be offered. Currently, rapid HIV testing is performed in community health centers [9], domestic violence shelters [10], emergency departments [11], large urban jails [12], pharmacies [13], and primary care offices [14]. Dental clinics represent another potential setting that offers the advantage of being able to reach a large proportion of the general US population. According to the CDC, in 2015, 64% of adults aged 18 to 64 years and 63% of adults aged 65 years and over had visited a dentist in the preceding year [15]. Additionally, dental care providers regularly screen their patients for manifestations of systemic diseases [16], and their training includes a thorough foundation in communicable diseases, which could establish them as potential providers of rapid HIV testing [17,18].

Previous research studies from Kansas City [19], Los Angeles [20], and New York City [21] have indicated a high acceptance of potentially receiving an HIV test among people attending dental clinics. Specifically, 73% of 150 respondents in the Kansas City study reported they would be willing to receive free HIV testing during their dental visit [19], 71% of 383 respondents in the Los Angeles study indicated being willing to receive HIV testing at their dentist’s office [20], and 72% of 426 respondents in the New York City study reported being willing to get tested for HIV in a dental care setting (85% preferred an oral fluid rapid test, 5% preferred a finger-stick blood rapid test, 9% preferred a venipuncture blood test) [21]. Each of these studies included convenience samples drawn from local clinics, and their results cannot be generalized to other cities in the United States. We are not aware of any studies that have assessed patient willingness to receive HIV testing in dental care settings at a national level.

Using a web-based sample of adult residents of the United States who reported an HIV-negative or unknown serostatus, we sought to assess the willingness to receive any type of HIV testing in dental care settings and describe variations across strata of demographic characteristics and dental care-seeking history. We also sought to evaluate independent associations of willingness to receive HIV testing in dental care settings with the extent to which providers were perceived as knowledgeable about HIV and how comfortable respondents felt discussing HIV with their dental care providers. Understanding these issues can help guide future HIV education programs and prevention efforts, particularly an exploration of the facilitators and barriers to offering HIV testing in nontraditional settings such as dentists’ offices.

## Methods

Participants were recruited using banner advertisements featured on social networking platforms (Facebook and Instagram) from December 2018 to February 2019. Recruitment was targeted toward user profiles of those aged 18 years or older and residents of the United States and its dependent areas. The advertisements included diverse images of patients and dental care providers in clinical settings, the study title (Project Viva), as well as the following call-to-action text: “Would you be willing to take an HIV test at your dentist’s office? Tell us on this short University of Michigan survey!” Individuals who clicked through the banner advertisements were directed to an informed consent page programmed in Qualtrics, a web-based survey platform, and those who consented were screened to determine eligibility. The eligibility criteria included being at least 18 years of age, currently residing in the United States or its dependent areas, and reporting HIV-negative or unknown serostatus. Eligible individuals were directed to a voluntary web-based survey, which had an estimated time to completion of 15 minutes. No monetary incentives were provided to the participants for completing our survey. Ethical approval for this study was obtained from the University of Michigan’s institutional review board (HUM00153814).

Demographic information collected from participants included their age, race and ethnicity, highest level of education, gender identity, sexual orientation, and relationship status. Those who were partnered were asked about whether they were in a closed relationship (ie, sex with outside partners was not allowed), an open relationship in which sex with outside partners was allowed with certain rules or restrictions, or an open relationship in which sex with outside partners was allowed without any rules or restrictions. State of current residence could be selected from a drop-down list, and this information was used to create regional categories (Northeast, Midwest, South, West). Participants were also asked about their sexual behaviors in the past 6 months (oral sex, vaginal sex, and anal sex), and their HIV testing history. Several questions were used to elicit information on participant’s use of and experiences with seeking dental care services. Dental insurance coverage was assessed using the question: “What type of dental insurance do you currently have?” (Response options: private/work-based insurance, school-based insurance, Affordable Care Act, Medicaid/Medicare, Veterans Administration benefits, some other insurance, I do not have dental insurance.) Frequency of visiting a dental care provider was assessed using the question: “How many times did you see a dental care provider in the past year?” (Response options: 0, 1, ≥2.) Location of seeking dental care services was assessed using the question: “Where do you usually seek dental care services?” (Response options: private practice/clinic, community dental clinic, dental school clinic, mobile dental clinic, some other location, I do not have a source of dental care.) Perception regarding whether one’s dental care provider was knowledgeable about HIV was assessed using a 4-point Likert item asking participants the extent to which they agreed or disagreed with the following statement: “The provider where I usually seek dental care services is knowledgeable about HIV.” (Response options: strongly agree, somewhat agree, somewhat disagree, strongly disagree.) Similarly, participants’ level of comfort around discussing HIV with their dental care provider was assessed using the following 4-point Likert item question: “How comfortable do you feel discussing HIV with your dental care provider?” (Response options: very comfortable, somewhat comfortable, somewhat uncomfortable, very uncomfortable.)

Willingness to receive any type of HIV test in a dental care setting was assessed using the following question: “Did you know there are multiple ways to test for HIV? These include: 1. A traditional HIV test performed on blood, drawn using a syringe; 2. A rapid HIV test performed on blood, collected from a finger prick; 3. A rapid HIV test performed on an oral fluid sample, collected by swabbing your gums. If any of these tests could be offered by a dental care provider, would you be willing to have one in a dental care setting?” (Response options: very willing, somewhat willing, somewhat unwilling, very unwilling.) For analytical purposes, participants’ responses to this question were combined to construct a dichotomous variable for our outcome of interest: Willing to receive any type of HIV testing in a dental care setting—yes or no. Participants who responded being very or somewhat willing to receive any type of HIV testing were also asked to indicate their most preferred of the three options.

Statistical analyses were conducted using SAS version 9.4 (SAS Institute Inc). Because of our primary focus on the willingness to receive HIV testing in dental care settings, we restricted our analyses to participants who reported having a source of dental care and answered this question. The demographic and behavioral characteristics of the sample were summarized using descriptive statistics. Factors independently associated with participants’ willingness to receive any type of HIV testing in a dental care setting (ie, oral fluid rapid testing, finger-stick blood rapid testing, or venipuncture blood testing) were identified using a multivariable logistic regression model. Estimated logit plots were produced to determine whether age, collected as a continuous measure, should be treated as a continuous or categorical variable. Because age demonstrated a nonlinear relationship with our outcome of interest (willingness to receive any type of HIV testing in a dental care setting), it was treated as a categorical measure. An examination of the condition indices and variance decomposition proportions did not reveal any collinearity problems. Results from the model are presented as adjusted odds ratios (aORs) with their 95% confidence intervals.

## Results

Overall, 680,290 advertising impressions (ie, the number of times an advertisement displayed on a user’s screen within Facebook or Instagram) resulted in 3849 link clicks (ie, the number of users who clicked on the advertisements) to the survey landing page over an 8-week period. Of these, 548 individuals provided informed consent, 509 of whom were eligible to participate. Our final analytical sample was restricted to 421 of 509 study participants who reported having a source of dental care and provided data on their willingness to receive an HIV test in a dental care setting. Excluded participants were similar in demographic and behavioral characteristics to those whose data were analyzed.

Table 1 summarizes the descriptive characteristics of the 421 participants. The majority were younger than 35 years (mean 33 years, median 29 years), non-Hispanic white, had an associate’s or bachelor’s degree, and identified as cisgender. Over half (221/421, 52.5%) reported their orientation as either homosexual/gay, bisexual, queer, or questioning/unsure. A total of 255 (60.6%) reported having a primary partner (described to the participants as “Someone you feel committed to above all others. You might call this person your boyfriend/girlfriend, partner, significant other, spouse, or husband/wife”). Of these, 207 (81.2%) reported being in a closed relationship, 36 (14.1%) reported being in an open relationship with certain rules or restrictions, and 11 (4.3%) reported being in an open relationship without any rules or restrictions (1 participant did not respond to this question). Our sample included participants residing all across the United States—108 (25.7%) in the Northeast, 72 (17.1%) in the Midwest, 129 (30.6%) in the South, and 112 (26.6%) in the West.

Regarding sexual activity in the past 6 months, 271 of 421 participants (64.4%) reported engaging in oral sex, 197 (46.8%) reported engaging in vaginal sex, and 136 (32.3%) reported engaging in anal sex. Regarding number of sexual partners, 104 of 421 participants (24.7%) that reported oral sex, 39 (9.3%) that reported vaginal sex, and 49 (11.6%) that reported anal sex did so with ≥2 partners. With respect to participants’ HIV testing history, two-thirds (284/421, 67.5%) reported having ever been tested for HIV, 147 (34.9%) of whom had been tested in the past year. Of the 421 participants, 137 (32.5%) had never been tested for HIV.

**Table 1:** Demographic and behavioral characteristics of study participants, United States, December 2018 to February 2019.

Characteristic		Willing to receive any type of HIV testing in a dental care setting		Total (n=421) n (%)
		Yes (n=326)^a^ n (%)	No (n=95)^b^ n (%)	
**Age in years^c^**				
	18-24	105 (32.21)	29 (30.53)	134 (31.83)
	25-34	132 (40.49)	26 (27.37)	158 ( 37.53)
	≥35	89 (27.30)	40 (42.11)	129 ( 30.64)
**Race/ethnicity**				
	Hispanic	20 (6.13)	6 (6.32)	26 (6.18)
	Non-Hispanic, nonwhite^d^	60 (18.40)	10 (10.53)	70 (16.63)
	Non-Hispanic, white	246 (75.46)	79 (83.16)	325 (77.22)
**Educational level**				
	Associate’s degree or lower^e^	129 (39.57)	30 (31.58)	159 (37.77)
	Bachelor’s degree	118 (36.20)	34 (35.79)	152 (36.10)
	Master’s degree or higher^f^	79 (24.23)	31 (32.63)	110 (26.13)
**Gender identity**				
	Cisgender male	128 (39.26)	40 (42.11)	168 (39.90)
	Cisgender female	171 (52.45)	49 (51.58)	220 (52.26)
	Other^g^	27 (8.28)	6 (6.32)	33 (7.84)
**Sexual orientation**				
	Heterosexual/straight	152 (46.63)	48 (50.53)	200 ( 47.51)
	Homosexual/gay	80 (24.54)	30 (31.58)	110 (26.13)
	Other^h^	94 (28.83)	17 (17.89)	111 (26.37)
**Relationship status**				
	Single	129 (39.57)	37 (38.95)	166 (39.43)
	Partnered^i^	197 (60.43)	58 (61.05)	255 (60.57)
**Region**				
	Northeast	83 (25.46)	25 (26.32)	108 (25.65)
	Midwest	56 (17.18)	16 (16.84)	72 (17.10)
	South	102 (31.29)	27 (28.42)	129 (30.64)
	West	85 (26.07)	27 (28.42)	112 (26.60)
**Engaged in oral sex in the past 6 months**				
	Yes, with ≥2 partners	86 (26.38)	18 (18.95)	104 (24.70)
	Yes, with 1 partner	132 (40.49)	35 (36.84)	167 (39.67)
	No	108 (33.13)	42 (44.21)	150 (35.63)
**Engaged in vaginal sex in the past 6 months**				
	Yes, with ≥2 partners	36 (11.04)	3 (3.16)	39 (9.26)
	Yes, with 1 partner	122 (37.42)	36 (37.89)	158 (37.53)
	No	168 (51.53)	56 (58.95)	224 (53.21)
**Engaged in anal sex in the past 6 months**				
	Yes, with ≥2 partners	38 (11.66)	11 (11.58)	49 (11.64)
	Yes, with 1 partner	67 (20.55)	20 (21.05)	87 (20.67)
	No	221 (67.79)	64 (67.37)	285 (67.70)
**HIV testing history**				
	Tested in past year	119 (36.50)	28 (29.47)	147 (34.92)
	Tested more than a year ago	101 (30.98)	36 (37.89)	137 (32.54)
	Never been tested	106 (32.52)	31 (32.63)	137 (32.54)

^a^Includes 204 who were very willing and 122 who were somewhat willing.

^b^Includes 53 who were somewhat unwilling and 42 who were very unwilling.

^c^Age: mean 33 years, median 29 years, range 18 to 73 years.

^d^Includes 27 non-Hispanic black/African American, 14 Asian, 4 Native American/Alaskan Native, 1 Middle Eastern/Arab American, 20 mixed, and 4 other.

^e^Includes 26 with an Associate’s degree, 110 with some college education, 19 with a high school diploma, 3 with some high school education, and 1 who never went to high school.

^f^Includes 84 with a Master’s degree and 26 with a doctoral degree.

^g^Includes 12 transgender male, 1 transgender female, 13 genderqueer/nonbinary, 5 gender fluid, and 2 other.

^h^Includes 68 bisexual, 25 queer, 11 questioning/unsure, and 7 other.

^i^Includes 207 who reported being in a closed relationship (ie, sex with outside partners was not allowed), 36 who reported being in an open relationship in which sex with outside partners was allowed with certain rules or restrictions, 11 who reported being in an open relationship in which sex with outside partners was allowed without any rules or restrictions, and 1 who did not respond to this question.

Table 2 summarizes the previous use of and experiences with seeking dental care services among our study participants. Most participants had dental insurance coverage (356/421, 84.6%), saw a dental care provider at least once in the past year (340/421, 80.8%), and usually sought dental care services at a private practice or clinic (388/421, 92.2%). Slightly over a third (162/421, 38.5%) strongly or somewhat agreed that their dental care provider was knowledgeable about HIV, and a smaller proportion (137/421, 32.5%) felt very or somewhat comfortable discussing HIV with their dental care provider.

Acceptability of receiving any kind of HIV testing in a dental care setting was high, with more than three-fourths being very or somewhat willing (326/421, 77.4%). Of these participants, most were between the ages of 18 and 34 years (237/326, 72.7%), cisgender female (171/326, 52.5%), homosexual/gay, bisexual, queer, or questioning/unsure (174/326, 53.4%), partnered (197/326, 60.4%), and had been tested for HIV at least once in their lifetime (220/326, 67.5%). Most of the participants who reported being very or somewhat willing to receive HIV testing in a dental care setting had dental insurance (276/326, 84.7%) and had seen a dental care provider at least once in the past year (261/326, 80.1%). The majority strongly or somewhat disagreed that their dental care provider was knowledgeable about HIV (184/326, 56.4%) and felt very or somewhat uncomfortable discussing HIV with their dental care provider (195/326, 59.8%). Regarding the type of HIV test that would be most preferred, 79.9% (259/326) reported they would prefer receiving an oral fluid rapid test, 9.9% (32/326) reported they would prefer receiving a finger-stick blood rapid test, and 10.2% (33/326) reported they would prefer receiving a venipuncture blood test (2 participants did not respond to this question).

Results from our multivariable logistic regression model used to identify factors independently associated with willingness to receive any type of HIV testing in a dental care setting are summarized in Table 3. Participants aged 18 to 24 years (aOR 3.22, 95% CI 1.48-6.98) and 25 to 34 years (aOR 5.26, 95% CI 2.52-10.98) were significantly more willing compared with those who were ≥35 years. Believing that one’s dental care provider was knowledgeable about HIV (aOR 2.04, 95% CI 1.06-3.92) and feeling comfortable discussing HIV with one’s dental care provider (aOR 9.84, 95% CI 3.99-24.27) were also positively associated with willingness to receive any type of HIV testing in a dental care setting. Given that 259 of 326 (79.9%) willing participants reported they would prefer receiving an oral fluid rapid test, we performed a sensitivity analysis using a subsample of 354 participants to identify factors independently associated with willingness to receive oral fluid rapid HIV testing in a dental care setting. The results of this multivariable logistic regression model were similar. Participants aged 18 to 24 years (aOR 3.48, 95% CI 1.54-7.85) and 25 to 34 years (aOR 5.57, 95% CI 2.58-12.03) were significantly more willing compared with those who were ≥35 years, as were non-Hispanic nonwhite participants (aOR 2.34, 95% CI 1.03-5.33) compared with non-Hispanic white participants. Feeling comfortable discussing HIV with one’s dental care provider (aOR 10.03, 95% CI 3.94-25.50) was also positively associated with willingness to receive oral fluid rapid HIV testing.

**Table 2:** Use of and experiences with seeking dental care services among study participants, United States, December 2018 to February 2019.

Characteristic		Willing to receive any type of HIV testing in a dental care setting		Total (n=421) n (%)
		Yes (n=326)^a^ n (%)	No (n=95)^b^ n (%)	
**Dental insurance coverage**				
	Insured^c^	276 (84.66)	80 (84.21)	356 (84.56)
	Uninsured	50 (15.34)	15 (15.79)	65 (15.44)
**Number of visits to a dental care provider in the past year**				
	≥2	177 (54.29)	48 (50.53)	225 (53.44)
	1	84 (25.77)	31 (32.63)	115 (27.32)
	0	65 (19.94)	16 (16.84)	81 (19.24)
**Usual location of seeking dental care services**				
	Private practice/clinic	296 (90.80)	92 (96.84)	388 (92.16)
	Other^d^	30 (9.20)	3 (3.16)	33 (7.84)
**Level of agreement regarding whether one’s dental care provider is knowledgeable about HIV^e^**				
	Strongly agree	44 (13.58)	2 (2.13)	46 (11.00)
	Somewhat agree	96 (29.63)	20 (21.28)	116 (27.75)
	Somewhat disagree	128 (39.51)	36 (38.30)	164 (39.24)
	Strongly disagree	56 (17.28)	36 (38.30)	92 (22.01)
**Level of comfort around discussing HIV with one’s dental care provider^f^**				
	Very comfortable	50 (15.38)	3 (3.16)	53 (12.62)
	Somewhat comfortable	80 (24.62)	4 (4.21)	84 (20.00)
	Somewhat uncomfortable	115 (35.38)	21 (22.11)	136 (32.38)
	Very uncomfortable	80 (24.62)	67 (70.53)	147 (35.00)

^a^Includes 204 who were very willing and 122 who were somewhat willing.

^b^Includes 53 who were somewhat unwilling and 42 who were very unwilling.

^c^Includes 293 with private/work-based insurance, 37 with Medicaid/Medicare, 9 with Affordable Care Act insurance, 8 with school-based insurance, 2 with Veterans Administration benefits, and 7 with some other insurance.

^d^Includes 15 at a community dental clinic, 14 at a dental school clinic, 1 at a mobile dental clinic, and 3 at some other location.

^e^Numbers do not add to total because 3 participants did not respond to this question.

^f^Numbers do not add to total because 1 participant did not respond to this question.

**Table 3:** Factors associated with willingness to receive any type of HIV testing in a dental care setting, United States, December 2018 to February 2019.

Characteristic		Willing to receive any type of HIV testing in a dental care setting, Adjusted odds ratio (95% CI)	*P* value
**Age in years^a^**			<.001
	18-24	3.22 (1.48-6.98)	
	25-34	5.26 (2.52-10.98)	
	≥35	referent	
**Race/ethnicity**			.07
	Hispanic	0.51 (0.17-1.56)	
	Non-Hispanic, nonwhite^b^	2.16 (0.96-4.83)	
	Non-Hispanic, white	referent	
**Educational level**			.12
	Associate’s degree or lower^c^	2.14 (1.00-4.52)	
	Bachelor’s degree	1.32 (0.67-2.61)	
	Master’s degree or higher^d^	referent	
**Gender identity**			.44
	Cisgender male	0.62 (0.19-2.00)	
	Cisgender female	0.95 (0.31-2.97)	
	Other^e^	referent	
**Sexual orientation**			.80
	Heterosexual/straight	0.78 (0.36-1.66)	
	Homosexual/gay	0.79 (0.31-2.97)	
	Other^f^	referent	
**Relationship status**			.84
	Single	1.06 (0.61-1.83)	
	Partnered^g^	referent	
**Engaged in oral, sex with ≥2 partners in the past 6 months**			.29
	Yes	1.73 (0.62-4.81)	
	No	referent	
**Engaged in vaginal sex with ≥2 partners in the past 6 months**			.40
	Yes	1.94 (0.42-9.05)	
	No	referent	
**Engaged in anal sex with ≥2 partners in the past 6 months**			.83
	Yes	0.88 (0.58-2.95)	
	No	referent	
**HIV testing history**			.82
	Tested in the past year	1.23 (0.58-2.58)	
	Tested more than a year ago	0.98 (0.50-1.92)	
	Never been tested	referent	
**Dental insurance coverage**			.87
	Insured^h^	0.95 (0.46-1.97)	
	Uninsured	referent	
**Number of visits to a dental care provider in the past year**			.25
	≥2	0.96 (0.46-2.00)	
	1	0.58 (0.26-1.31)	
	0	referent	
**Agree that one’s dental care provider is knowledgeable about HIV**			.03
	Yes^i^	2.04 (1.06-3.92)	
	No^j^	referent	
**Feel comfortable discussing HIV with one’s dental care provider**			<.001
	Yes^k^	9.84 (3.99-24.27)	
	No^l^	referent	

^a^Age: mean 33 years, median 29 years, range 18 to 73 years.

^b^Includes 27 non-Hispanic black/African American, 14 Asian, 4 Native American/Alaskan Native, 1 Middle Eastern/Arab American, 20 mixed, and 4 other.

^c^Includes 26 with an Associate’s degree, 110 with some college education, 19 with a high school diploma, 3 with some high school education, and 1 who never went to high school.

^d^Includes 84 with a Master’s degree and 26 with a doctoral degree.

^e^Includes 12 transgender male, 1 transgender female, 13 genderqueer/nonbinary, 5 gender fluid, and 2 other.

^f^Includes 68 bisexual, 25 queer, 11 questioning/unsure, and 7 other.

^g^Includes 207 who reported being in a closed relationship (ie, sex with outside partners was not allowed), 36 who reported being in an open relationship in which sex with outside partners was allowed with certain rules or restrictions, 11 who reported being in an open relationship in which sex with outside partners was allowed without any rules or restrictions, and 1 who did not respond to this question.

^h^Includes 293 with private/work-based insurance, 37 with Medicaid/Medicare, 9 with Affordable Care Act insurance, 8 with school-based insurance, 2 with Veterans Administration benefits, and 7 with some other insurance.

^i^Includes 46 who strongly agree and 116 who somewhat agree.

^j^Includes 92 who strongly disagree and 164 who somewhat disagree.

^k^Includes 53 who feel very comfortable and 84 who feel somewhat comfortable.

^l^Includes 147 who feel very uncomfortable and 136 who feel somewhat uncomfortable.

## Discussion

### Principal Findings

Our study found high levels of willingness to receive any type of HIV testing (ie, oral fluid rapid testing, finger-stick blood rapid testing, or venipuncture blood testing) in a dental care setting across selected strata of web-using adults in the United States. Higher levels of willingness were associated with being younger than 35 years, believing that one’s dental care provider is knowledgeable about HIV, and feeling comfortable discussing HIV with one’s dental care provider. Given that more than three-fourths of our sample expressed a favorable attitude toward this approach, a third of whom had never been tested for HIV, it might be useful to explore whether HIV prevention efforts could be expanded to include dental care settings and optimal ways of potentially initiating the process. Our results also suggest that efforts to improve patients’ perceptions of whether their dental care providers are knowledgeable about HIV and their comfort levels around discussing HIV could be an important consideration for successfully implementing HIV testing in this nontraditional setting.

First, we focus our discussion on the demographic characteristics associated with willingness to receive any type of HIV testing in a dental care setting. Participants aged 18 to 24 years were more than 3 times as likely to report being willing and those aged 25 to 34 years were more than 5 times as likely to report being willing compared with those aged ≥35 years. Although not statistically significant at an α level of .05, non-Hispanic, nonwhite participants were twice as likely to report being willing to receive any type of HIV test in a dental care setting compared with non-Hispanic white participants. However, this association was significant in the subsample of 354 participants in which we examined associations with willingness to receive oral fluid rapid HIV testing. These results are important in light of the disproportionate burden of HIV among people under the age of 35 years and black/African American individuals [22]. The latest CDC models estimate that 44% of HIV-positive individuals younger than 25 years and 29% of HIV-positive individuals aged 25 to 34 years are unaware of their serostatus [23]. In addition, 15% of blacks/African Americans currently living with HIV are unaware of their infection [23]. This is despite the fact that in 2017, the majority of CDC-funded HIV tests were received by people in their twenties and thirties, and black/African American individuals got tested at much higher rates than any other racial/ethnic group [24]. Collectively, our results indicate that although not everyone would be willing to undergo HIV testing at their dental care providers’ offices, certain high-risk subgroups may benefit from this strategy.

Turning to focus on how patients’ perceptions of dental care provider knowledge about HIV and their comfort levels around discussing HIV with their dental care providers might influence willingness to receive HIV testing, our study found some noteworthy associations. Participants who believed that their dental care provider was knowledgeable about HIV were more than twice as likely to report being willing to receive any type of HIV testing in a dental care setting versus those who did not. Only 38% of our sample believed that their dental care provider was knowledgeable about HIV. Participants who felt comfortable discussing HIV with their dental care provider were approximately 10 times as likely to report being willing to receive any type of HIV testing in a dental care setting versus whose who did not. Only 33% of our sample felt comfortable discussing HIV with their dental care provider. These findings highlight the role that constructs such as perceived knowledge and comfort around discussing sensitive topics could play in influencing health promoting behaviors. Typically, patients do not engage in conversations about their sexual health with dental care providers, which might negatively influence their perceptions about provider knowledge pertaining to HIV prevention and treatment. Individuals are also unlikely to be aware of the nature and extent of HIV-related training received by dental care providers. In a qualitative study involving 19 attendees of a low-cost dental clinic in New York City, participants raised concerns about the negative psychological impact a positive HIV test result could have not just on patients, but also on providers who might not be trained to handle the situation [25]. Approximately a third of these participants stated they believed there was a need for professional counseling and linkage to care for anyone testing HIV positive. Informing patients that dental care providers receive education on HIV (as oral lesions are one of the first overt clinical features of infection) and are skilled in delivering potentially concerning news to facilitate referral for appropriate management (eg, in the case of suspected oropharyngeal cancer) might help improve perceptions. Good interpersonal and communication skills in dentistry are known to foster a positive provider-patient relationship [26,27]. Active communication strategies, assessing the emotional states of patients, and demonstrating empathy might help create rapport and a trusting relationship between dental care providers and their patients.

Our findings pertaining to patients’ perceptions of provider knowledge about HIV and their comfort levels around discussing HIV along with the literature on dental care providers’ attitudes toward and knowledge of HIV testing highlight several challenges to a potential large-scale implementation of HIV testing in dental care settings. One large national study that assessed 1802 dentists’ attitudes toward HIV testing found that only 57% were willing to offer HIV testing and even fewer (40%) believed that HIV testing is part of their role [28]. Only 14 participants in that study reported they were currently offering HIV testing, and less than 1 in 8 were familiar with the CDC guidelines that recommend routine HIV screening of US adults in outpatient health care settings [3]. Nonetheless, recent qualitative research with dentists who are currently offering HIV testing lends support for the notion that dental care providers could take an expanded role in patients’ overall well-being. Participants in one study recognized the public health value in identifying undiagnosed persons in a timely manner and linking them to medical care and believed that dentists should start functioning as “total health providers, not just providers of the mouth” [29]. However, they also cited several barriers to the mainstream incorporation of HIV testing into dental care settings. These include the lack of an American Dental Association reimbursement code, perceived time constraints, feeling ill-equipped to deliver positive HIV test results, and concerns about the appropriateness of HIV testing in dentistry, a profession that has historically been characterized as generating more fear and anxiety than other forms of health care.

### Strengths and Limitations

Strengths of our study include examining variations in the willingness to receive any type of HIV testing in a dental care setting across demographic strata of a diverse sample of adults recruited from across the United States. The use of social media platforms allowed us to collect data in a time- and resource-efficient manner from a large number of individuals who had not visited a dental care provider in the past year (81/421, 19.2%). Our survey was voluntary and could only be accessed by clicking on our banner advertisements, so it is unlikely that the same individual would have responded more than once. Because the web might offer opportunities to create and maintain networks of relationships from which people could potentially draw health and social support resources [30], it is important to explore how to best harness its full potential to improve people’s health, particularly with regard to HIV testing. Notably, over half of our sample identified as either gay, bisexual, queer, or questioning, which is considerably higher than the proportion of these demographic subgroups in the general US population [31]. Given that sexual and gender minorities are disproportionately impacted by the HIV epidemic [32], it is encouraging that individuals responded to our Facebook and Instagram advertisements that suggested the potential for HIV testing in a nontraditional setting.

However, we acknowledge that our study is not without limitations. Our convenience sampling process yielded a group that was predominately non-Hispanic white. The underrepresentation of racial and ethnic minorities in our sample is comparable to previous web-based research studies [33]. The majority of our participants had dental insurance coverage, and more than three-fourths saw a dental care provider at least once in past year. These estimates are higher than the most recent available data from the US National Center for Health Statistics, in which only 50% adults aged 18 to 64 years had dental insurance coverage [34] and only 64% had visited a dental care provider in the past year [35]. Therefore, our results cannot be generalized to adults across the country. Finally, recent data management and security concerns at Facebook might have influenced the extent to which potential or actual participants felt comfortable responding to questions from an online source regarding their personal health information [36].

### Conclusions

Despite these limitations, our study adds to the growing body of literature on the willingness to receive HIV testing in dental care settings. Thus far, the data suggest that patients are highly willing to receive HIV testing (particularly an oral fluid rapid test), and in our study, this willingness varied across age, perceived dental care provider knowledge of HIV, and comfort levels discussing HIV with providers. Novel strategies are needed to encourage adults in the United States who might be at risk for acquiring HIV to discuss their risk with providers in all outpatient care settings. Future research should focus on assessing dental care providers’ attitudes, self-efficacy, and beliefs about whether HIV testing fits into the scope of dentistry. Trainings and interventions to enhance dental care provider education, including those that incorporate evidence-based practices in HIV prevention such as patient-centered care, harm reduction, and motivational interviewing [37,38], could prove beneficial.

## Abbreviations

aOR: adjusted odds ratio
CDC: US Centers for Disease Control and Prevention
FDA: US Food and Drug Administration
